# Supplementary material for: Quantifying local malignant adaptation in tissue‐specific evolutionary trajectories by harnessing cancer’s repeatability at the genetic level
Source: Evol Appl. 2019 Mar 18;12(5):1062–75. doi: 10.1111/eva.12781 (PMC6503823; doi:10.1111/eva.12781)
Supplement: Supplementary file 1 [file EVA-12-1062-s001.docx]

Supplementary Information

# Supplementary Methods

**Quantifying Alteration Self-sufficiency**

To calculate self-sufficiency, the number of “partners” of each driver alteration was first computed. For each driver alteration *d* in a tumour type, this is given by the number of additional clonal drivers in *S_d_*, the subset of samples in which *d* is clonally mutated. For *d*, *Np_d_* is thus a vector of number of partners of the same size as *S_d_*. Defining *Np_nd_* as the number of partners of all other drivers (“non *d*”) in all the samples in which they are mutated, we then compute *P_d_*, the power of finding such difference in mean between *Np_d_* and *Np_nd_* using the power.t.test R function, with *Np_nd_* as the reference distribution. As self-sufficiency is inversely correlated to the number of partners, *SS_d_* the self-sufficiency ratio for *d* is given by the following equation:

1. ${SS}_{d} ={(\frac{mean({Np}_{nd})}{mean({Np}_{d})})}^{P_{d}}$

SS*_d_* is thus centred around 1, increases if *d* has fewer partners than the other drivers, and the deviation from 1 is lessened when statistical power is low due to few observations for *d*. In order to avoid infinite ratios when the mean of *Np_d_* is 0, due to the absence of any partner in all samples where d is mutated, *mean(NP_d_)* is substituted by (1 / (*length(Np_d_)* + 1)) in these cases. Only three such cases occurred, all in kidney clear cell carcinoma (KIRC) with ELF1, HDAC9 and SHMT1 mutations.

**Quantifying Malignant Epistatic Interactions**

The malignant epistatic interactions we quantify in this article should be viewed more as an indicator of the consequence of these interactions on malignant transformation (we see these alterations co-occur more/less often than expected after selection/adaptation took place), that their actual contribution to fitness (the genotype grows in the pop more/less than predicted by individual contributions).

We consider *n*, the total number of clonal alterations across all patients in cohort for a given tumour type, two genomic alterations *A* and *B* with respective numbers of occurrences, and a sample *s* harbouring a specific number of alterations *x*. For any given sample *s*, the probability that it presents an alteration in *A* (or *B*) is therefore a hypergeometric problem, akin to drawing *x* balls from a total of *n*, with a rate of success depending on how many *A* (or *B*) alterations are present in a population. Let’s consider the case where *n*, the total number of alterations in the cohort, is 1000; 50 of these alterations correspond to *A* and 25 correspond to *B*; sample *s* harbours *x*=20 alterations.

We use the R phyper function to compute the probability of observing 1 or more *A* alterations in *s* (>0, hence the lower.tail=F and q=0 parameters), as follows :

p(*A*) = phyper(m=50, n=(1000-50), k=20, q=0, lower.tail=F) = 0.65

Similarly, the probability of observing 1 or more B alterations in sample s is calculated as follows:

P(*B*) = phyper(m=25, n=(1000-25), k=20, q=0, lower.tail=F) = 0.40

By assuming that these two events are independent, the probability that *s* harbours both alterations is calculated by the following formula:

P(*A*&*B*) = P(*A*) ∙ P(*B*) = 0.65 x 0.40 = 0.26.

Similarly, in a sample with 5 alterations, P(*A*) = 0.23; P(*B*) = 0.12 and thus P(*A*&*B*) = 0.03. This methodology incorporates the impact of the number of alterations in each sample, modeling that co-occurrence is more likely in hypermutated samples and thus being an improvement over a simple number of *A*/*n* approach, by.

By calculating the probability of co-occurrence across all samples of a cohort, based on the respective alteration load of each sample, we obtained a vector of P(*A*&*B*) probabilities (**Supplementary Methods Figure 1a**). We use this vector as a per-sample distribution to estimate the expected number of co-occurrences for *A* and *B* using a random draw procedure consisting of 10,000 iterations. In each iteration, we simulate whether we draw a “success” (*A* and *B* co-occur) in each sample, based on the individual P(*A*&*B*) of each sample to determine the probability of success. For each simulation, we therefore obtain a number of samples of the cohort in which *A* and *B* virtually co-occurred, based on the sample-specific probabilities. The 10,000 simulations thus result in a null distribution of what to expect for the number of times *A* and *B* are supposed to co-occur in the cohort (**Supplementary Methods Figure 1b**). The illustration bellows corresponds to the real case of BRAF (139/103155 alterations) and NRAS mutations (67/1031555 alterations) in the melanoma (SKCM) TCGA dataset (248 samples, each harbouring 416 ± 737 alterations).


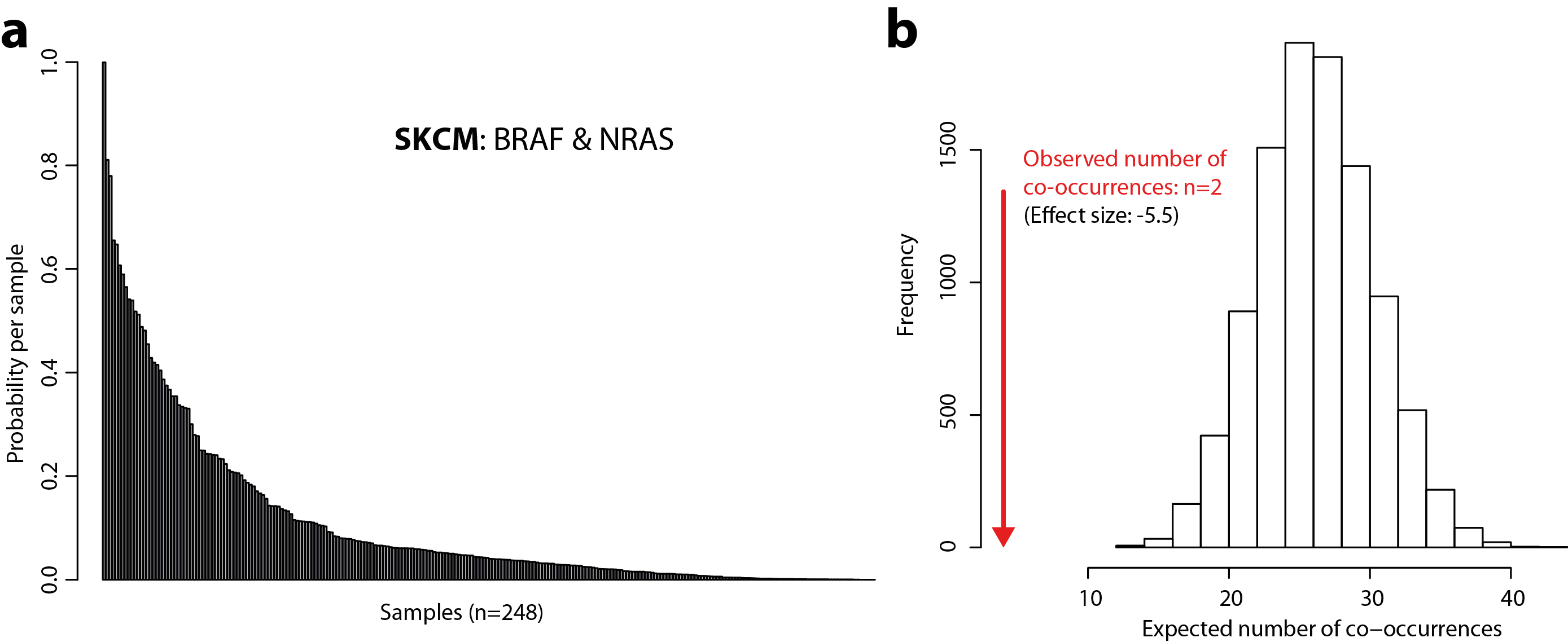


**Supplementary Methods Figure 1 – BRAF and NRAS co-occurrence in melanoma samples.** a) Sorted probabilities that NRAS and BRAF are co-occurring in each sample, based on sample-specific hypergeometric tests. b) Distribution of the number of expected co-occurrences according to 10,000 draws. In red is highlighted the actual number of samples in which NRAS and BRAF co-occur in the data.

If the observed number of co-occurrences was 0, it was set to either 0.5 if the mean of all draws was > 0.5, or to the mean of the draws otherwise, so that the ratio would be 1 (i.e. the interaction is neither positive nor negative due to lack of sufficient observations). For each interaction we calculated *R_ab_*, the ratio of observed occurrences divided by the mean expected occurrences. We weighted the final score *E_ab_* so as to penalise the few outliers observed when two very infrequent genes co-occur potentially by chance. This was done by bringing *R_ab_* closer to 1 for rare alterations thanks to the following formula, where (*A* U *B*) is the number of samples harbouring either *A* or *B* and *N* the total number of samples:

1. $E_{ab} = 1 - (\left( 1 - R_{ab} \right)\times\frac{\left( A\cup B \right)}{N})$

# Supplementary Tables

**Supplementary Table 1 – Model formulas.** Formulas used to calculate the contribution of each driver to a given sample’s Local Malignant Adaptation score. **Fa**: weighting factor for all selective advantages **SA** ; **Fs**: weighting factor for all self-sufficiency scores **SS** ; **Fi**: weighting factor for all intrinsic properties (**SA** x **SS**) ; **Fe**: weighting factor for all epistatic interactions **E**. **E*_d_*** corresponds to all interactions between driver *d* and the other clonally mutated drivers in a given sample. prod(E*_d_*) corresponds to the product of all elements of E*_d_* ; mean(E*_d_*) corresponds to the mean of all elements of E*_d_*.

| **Model name** | **Formula for driver *d*** |
| --- | --- |
| separate_mean | (Fa * SA*_d_*) + (Fs * SS*_d_*) + (Fe * mean(E*_d_*)) |
| separate_prod | (Fa * SA*_d_*) + (Fs * SS*_d_*) + (Fe * prod(E*_d_*)) |
| combined_mean | (Fi * SA*_d_* * SS*_d_*) + (Fe * mean(E*_d_*)) |
| combined_prod | (Fi * SA*_d_* * SS*_d_*) + (Fe * prod(E*_d_*)) |

**Supplementary Table 2 – Primary tumour type definition.** The TCGA tumour type, or “set”, used to classify each sample (right column) according to the reported primary site in the MET500 original publication (left column).

| **Reported MET500 primary tumour type** | **Assigned TCGA set** |
| --- | --- |
| Colon Adenocarcinoma | COAD |
| Cutaneous Melanoma | SKCM |
| Solitary Fibrous Tumor | NA |
| Extrahepatic Cholangiocarcinoma | NA |
| Prostate Adenocarcinoma | NA |
| Dedifferentiated Liposarcoma | NA |
| Prostate Neuroendocrine Carcinoma | NA |
| Bladder Urothelial Carcinoma | BLCA |
| Thymic Neuroendocrine Tumor | NA |
| Extraskeletal Myxoid Chondrosarcoma | NA |
| Osteoblastic Osteosarcoma | NA |
| Embryonal Rhabdomyosarcoma | NA |
| Undifferentiated Pleomorphic Sarcoma | NA |
| Adrenocortical Carcinoma | NA |
| Breast Invasive Lobular Carcinoma | BRCA |
| Breast Invasive Ductal Carcinoma | BRCA |
| Anal Squamous Cell Carcinoma | NA |
| Atypical Lung Carcinoid | NA |
| Gastrointestinal Stromal Tumor | NA |
| Desmoplastic Small-Round-Cell Tumor | NA |
| Intrahepatic Cholangiocarcinoma | NA |
| Salivary Clear Cell Carcinoma | NA |
| Adenoid Cystic Carcinoma | NA |
| Pancreatic Adenocarcinoma | NA |
| Penile Squamous Cell Carcinoma | NA |
| Ewing Sarcoma | NA |
| Bladder Squamous Cell Carcinoma | BLCA |
| Serous Ovarian Cancer | NA |
| Anaplastic Thyroid Cancer | NA |
| Cutaneous Squamous Cell Carcinoma | NA |
| Follicular Dendritic Cell Sarcoma | NA |
| Pancreatic Neuroendocrine Tumor | NA |
| High-Grade Serous Ovarian Cancer | NA |
| Thymoma | NA |
| Ameloblastoma | NA |
| Renal Clear Cell Carcinoma | KIRC |
| Esophageal Adenocarcinoma | NA |
| Rectal Adenocarcinoma | COAD |
| Poorly Differentiated Carcinoma, NOS | NA |
| Dermatofibrosarcoma Protuberans | NA |
| Poorly Differentiated Thyroid Cancer | NA |
| Sarcomatoid Carcinoma | NA |
| Undifferentiated Malignant Neoplasm | NA |
| Solid Pseudopapillary Neoplasm of the Pancreas | NA |
| Small Cell Bladder Cancer | BLCA |
| Mucinous Adenocarcinoma | NA |
| Leiomyosarcoma | NA |
| Thymic Squamous Carcinoma | NA |
| Head and Neck Mucosal Melanoma | SKCM |
| Lung Adenocarcinoma | LUAD |
| Merkel Cell Carcinoma | NA |
| Pancreatobiliary Ampullary Carcinoma | NA |
| Hepatocellular Carcinoma | NA |
| Poorly Differentiated Non-Small Cell Lung Cancer | NA |
| Mucinous Cystic Neoplasm | NA |
| Synovial Sarcoma | NA |
| Fibrolamellar Carcinoma | NA |
| Uterine Leiomyosarcoma | NA |
| Squamous Cell Carcinoma, NOS | NA |
| Clear Cell Carcinoma | NA |
| Alveolar Soft Part Sarcoma | NA |
| Teratoma with Malignant Transformation | NA |
| Adenocarcinoma, NOS | NA |
| Lung Squamous Cell Carcinoma | LUSC |
| Oral Cavity Squamous Cell Carcinoma | HNSC |
| Low-Grade Serous Ovarian Cancer | NA |
| Urachal Adenocarcinoma | NA |
| Undifferentiated Pleomorphic Sarcoma/Malignant F | NA |
| Clear Cell Carcinoma of the Lung | NA |
| Renal Cell Carcinoma | KIRC |
| Urethral Urothelial Carcinoma | NA |
| Angiosarcoma | NA |
| Skin Adnexal Carcinoma | NA |
| Myxoid/Round-Cell Liposarcoma | NA |
| Stomach Adenocarcinoma | NA |
| Mullerian Poorly Differentiated Carcinoma | NA |
| Chromophobe Renal Cell Carcinoma | NA |
| Small Cell Carcinoma of Unknown Primary | NA |
| Perivascular Epithelioid Cell Tumor | NA |
| Neuroendocrine Carcinoma of the Stomach | NA |
| Inflammatory Breast Cancer | BRCA |
| Yolk Sac Tumor | NA |
| Oropharynx Squamous Cell Carcinoma | HNSC |
| Soft Tissue Myoepithelial Carcinoma | NA |
| Myxoid Chondrosarcoma | NA |
| Embryonal Carcinoma | NA |
| Signet Ring Cell Carcinoma of the Stomach | NA |
| Salivary Duct Carcinoma | NA |
| Granular Cell Tumor | NA |
| Mullerian Adenocarcinoma | NA |
| High-Grade Sarcoma | NA |
| Esophageal Squamous Cell Carcinoma | HNSC |
| Adamantinoma | NA |
| Metaplastic Breast Cancer | BRCA |
| Ovarian Adenocarcinoma | NA |
| Glioblastoma Multiforme | GBM |
| Myxofibrosarcoma | NA |
| Rhabdomyosarcoma | NA |
| Myoepithelial Carcinoma | NA |
| Sex Cord Stromal Tumor | NA |
| Head and Neck Neuroendocrine Carcinoma | NA |
| Adenosarcoma | NA |
| Neuroendocrine Tumor, NOS | NA |
| Collecting Duct Renal Cell Carcinoma | NA |
| Osteosarcoma | NA |
| Salivary Carcinoma | NA |
| Salivary Carcinoma, Other | NA |
| Cervical Neuroendocrine Tumor | NA |
| Breast Invasive Carcinoma, NOS | BRCA |
| Uterine Endometrioid Carcinoma | NA |
| Undifferentiated High-Grade Sarcoma | NA |
| Small Cell Lung Cancer | NA |
| Sebaceous Carcinoma | NA |
| Seminal Vesicle Carcinoma | NA |
| Atypical Meningioma | NA |
| Extramammary Paget Disease | NA |
| Prostate Carcinosarcoma | NA |
| Clear Cell Ovarian Cancer | NA |
| Pleural Mesothelioma | NA |
| Dedifferentiated Chondrosarcoma | NA |
| Ependymoma | NA |
| Sinonasal Squamous Cell Carcinoma | HNSC |

**Supplementary Table 3 – Tumour type corresponding to metastatic location.** The TCGA tumour type, or “set”, used to classify each sample (right column) according to the reported metastatic site in the MET500 original publication (left column).

| **Reported MET500 metastatic location** | **Assigned TCGA set** |
| --- | --- |
| Liver | NA |
| Skin | SKCM |
| Dura | NA |
| Shoulder | NA |
| Subq Nodule | NA |
| Lymph Node | NA |
| Lung | LUAD/LUSC |
| Chest Wall | NA |
| Subq Abdom. Mass | NA |
| Forearm Mass | NA |
| Retroperitoneum | NA |
| Pancreas | NA |
| Breast | BRCA |
| Bone | NA |
| Gluteus Muscle | NA |
| Thyroid | NA |
| Adrenal | NA |
| Neck | HNSC |
| Vertebra | NA |
| Esophagus | NA |
| Buttock | NA |
| Colon | COAD |
| Mandible | HNSC |
| Periaortic Mass | NA |
| Abdom. Wall | NA |
| Thoracic Epidural | NA |
| Paraspinal Mass | NA |
| Cheek | HNSC |
| Brain | GBM |
| Bladder | BLCA |
| Epidural | NA |
| Rectus Muscle | NA |
| Pelvis | NA |
| Pelvic Mass | NA |
| Peritoneum | NA |
| Pleura | NA |
| Abdomen | NA |
| Gluteal Mass | NA |
| Subq Nodule (Arm) | NA |
| Peritoneal Fluid | NA |
| Subcut. Abdomen | NA |
| Subcut. Periumbilical | NA |
| Acetabulum | NA |
| Lung Nodule | NA |
| Perirectal Mass | NA |
| Thigh | NA |
| Chest Wall Nodule | NA |
| Parotid | NA |
| Omentum | NA |
| Hilum | NA |
| Abdom. Nodule | NA |
| Skin Punch | SKCM |
| Sternal Mass | NA |
| Infraclavicular Mass | NA |
| Abdom. Mass | NA |
| Subq | NA |
| Neck Mass | HNSC |
| Subq Chest Wall | NA |
| Shoulder Mass | NA |
| Pleural Mass | NA |
| Back | NA |
| Thigh Mass | NA |
| Scapular Mass | NA |
| Submandible | HNSC |
| Mesenteric Mass | NA |
| Peritoneal Nodule | NA |
| Psoas Muscle | NA |
| Thoracic Fluid | NA |
| Hip Lesion | NA |
| Axillary Mass | NA |
| Omental Mass | NA |
| Cervix | NA |
| Spinal Mass | NA |
| Lacrimal Gland | NA |
| Prostate | NA |
| Arm | NA |
| Sphenoid Sinus | NA |

# Supplementary Figures


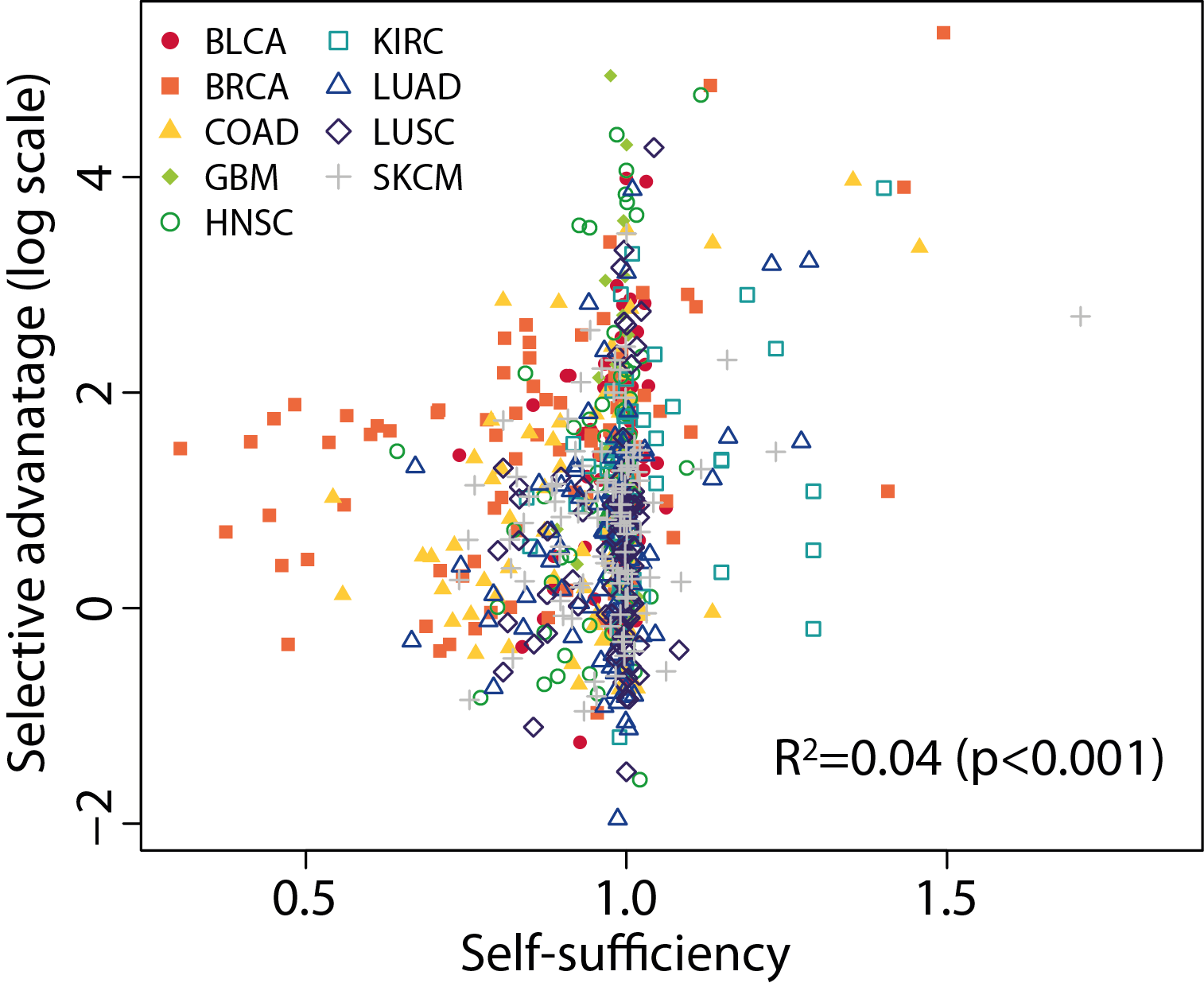


**Supplementary Figure 1 -** **Correlation between selective advantage and self-sufficiency**. Parameters are calculated across 9 tumour types, each identified by a different colour / symbol.


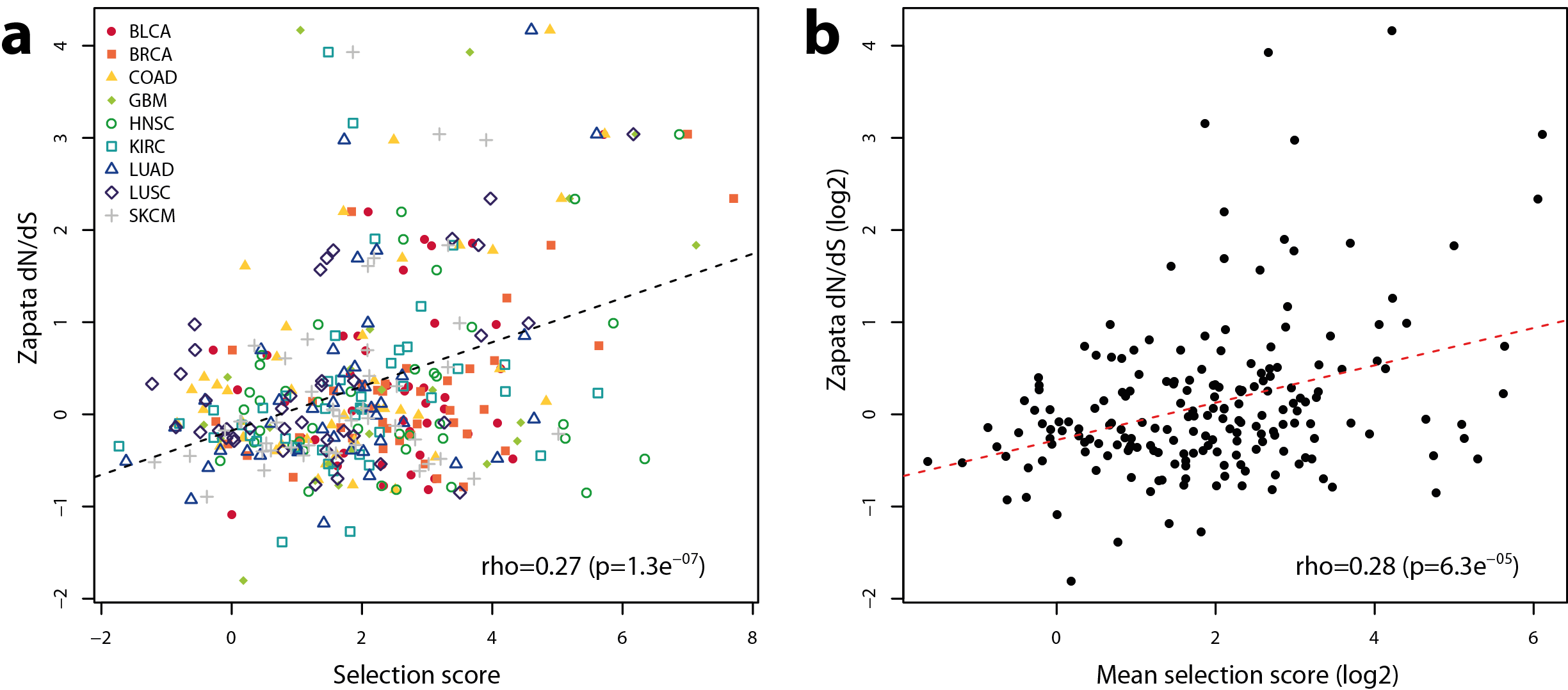


**Supplementary Figure 2 – Comparing selection score to a published corrected dN/dS.** a) Driver alterations per tumour type pooled together. For each tumour type, we included the top 50 genes from our calculations for which the corrected dN/dS was available in the Zapata et al. publication. Only gene mutations are therefore present, as copy number alterations are not suited to dN/dS and were not investigated by Zapata et al. Genes can be present multiple times as they can be drivers in multiple tumour types. Although our selection score will vary across tumour types, the dN/dS score is fixed since it is calculated on a pan-cancer basis. b) Average selection score. All genes that were in the top 50 drivers of at least one tumour type are present. If a gene was included in multiple tumour types, the mean of all relevant tumour types was computed. Each gene is thus represented only once. Spearman’s rank correlation coefficient rho was computed in both panels.


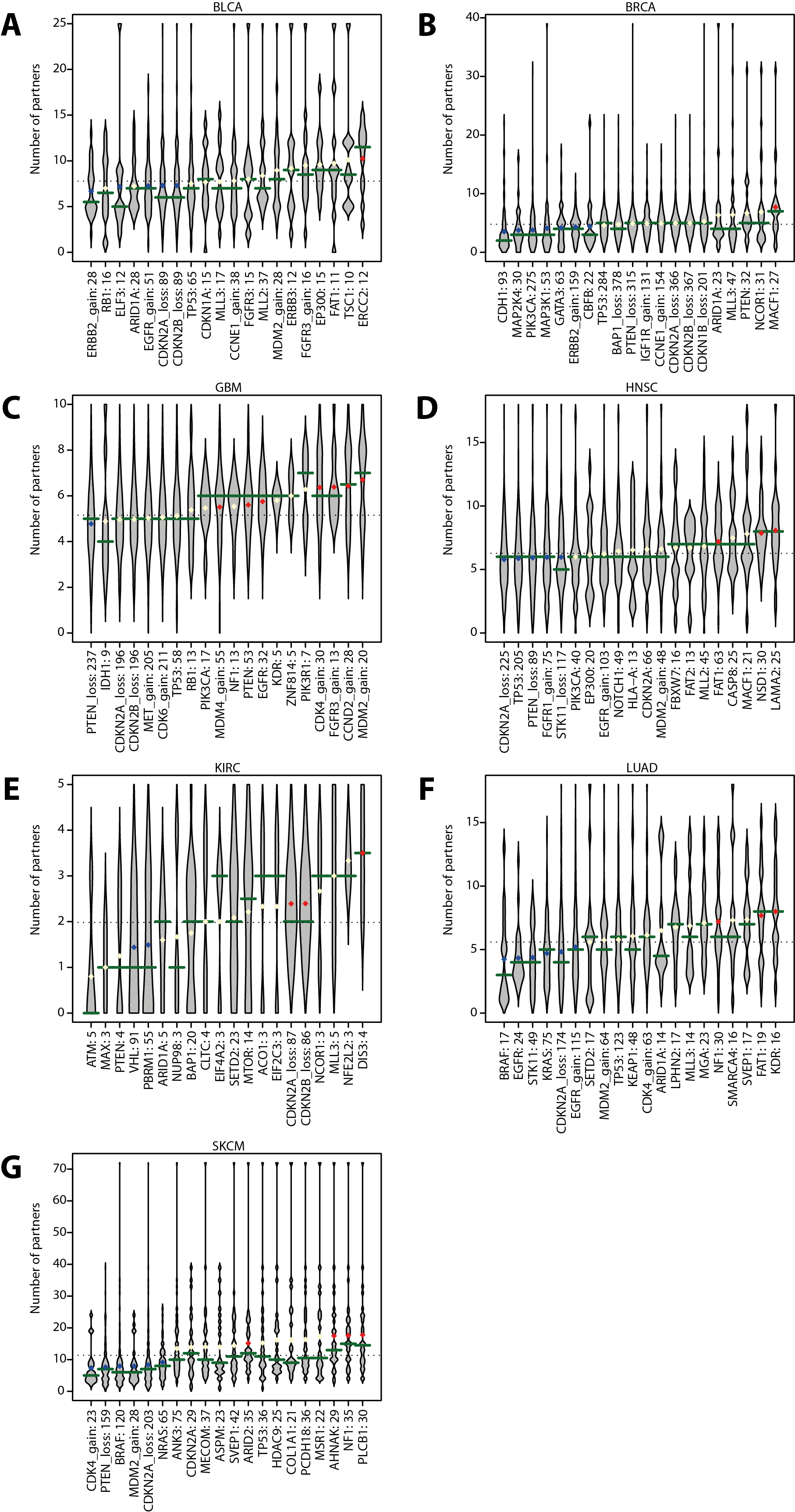


**Supplementary Figure 3 – Self-sufficiency and number of partners.** Number of additional drivers observed in samples harbouring the 20 most frequent alterations. Horizontal green bars represent the median, diamonds represent the mean. Blue means significantly fewer partners than expected, red means significantly more. Dotted line indicates the overall mean. A) Bladder Urothelial Carcinoma (BLCA). B) Breast invasive carcinoma (BRCA). C) Glioblastoma multiforme (GBM). D) Head and Neck squamous cell carcinoma (HNSC). E) Kidney renal clear cell carcinoma (KIRC). F) Lung adenocarcinoma (LUAD). G) Skin Cutaneous Melanoma (SKCM).


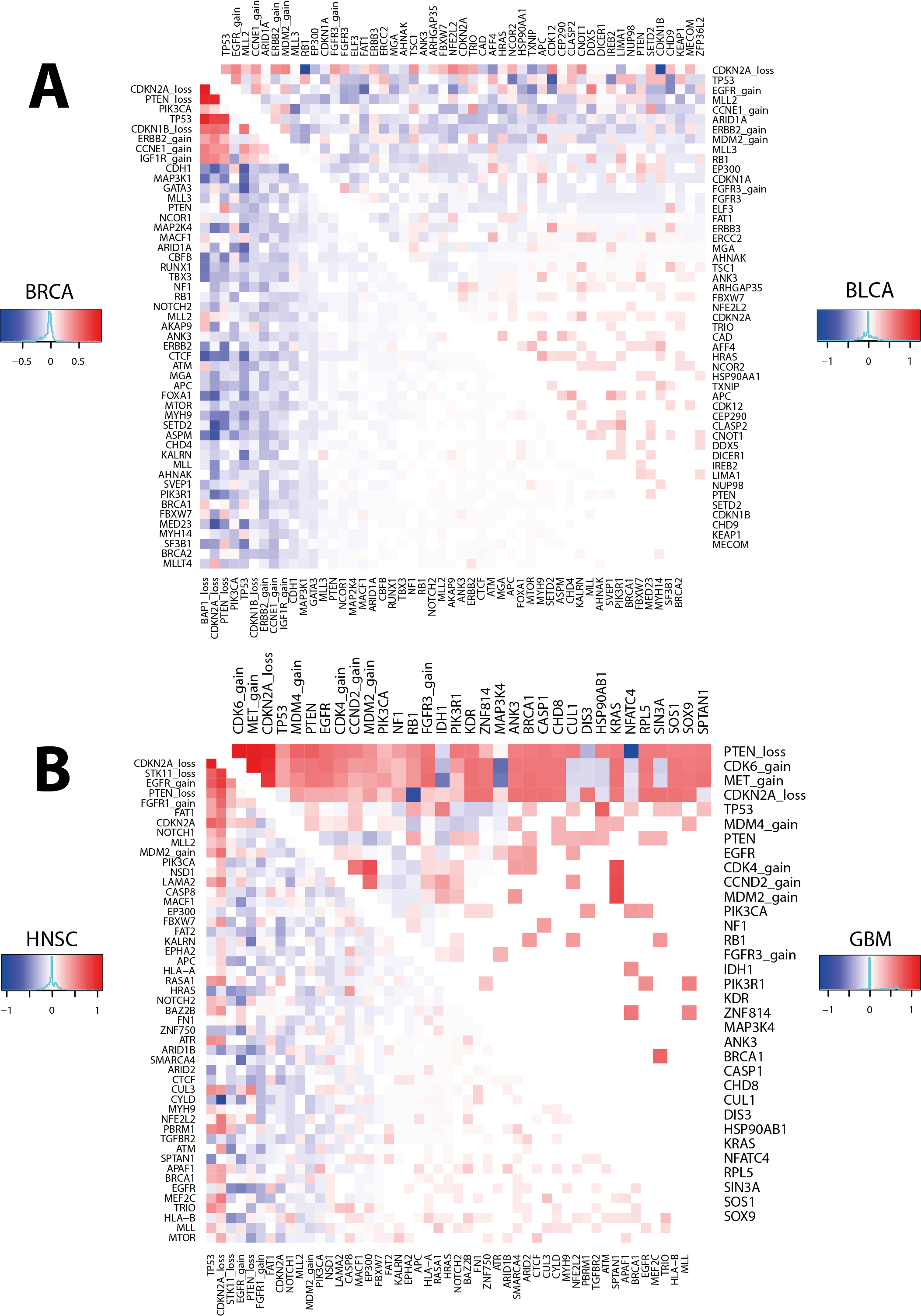


**Supplementary Figure 4 – Epistatic interactions.** Epistatic interactions between all retained drivers in different tumour types. Each tumour type corresponds to a half matrix. Blue indicates negative interaction due to co-occurrences rarer than expected, red indicates positive interactions. A) BLCA (top right) & BRCA (bottom left) ; B) GBM (top right) and HNSC (bottom left) ; C) KIRC (top right) and LUAD (bottom left) ; D) LUSC. *Continued on the next page.*


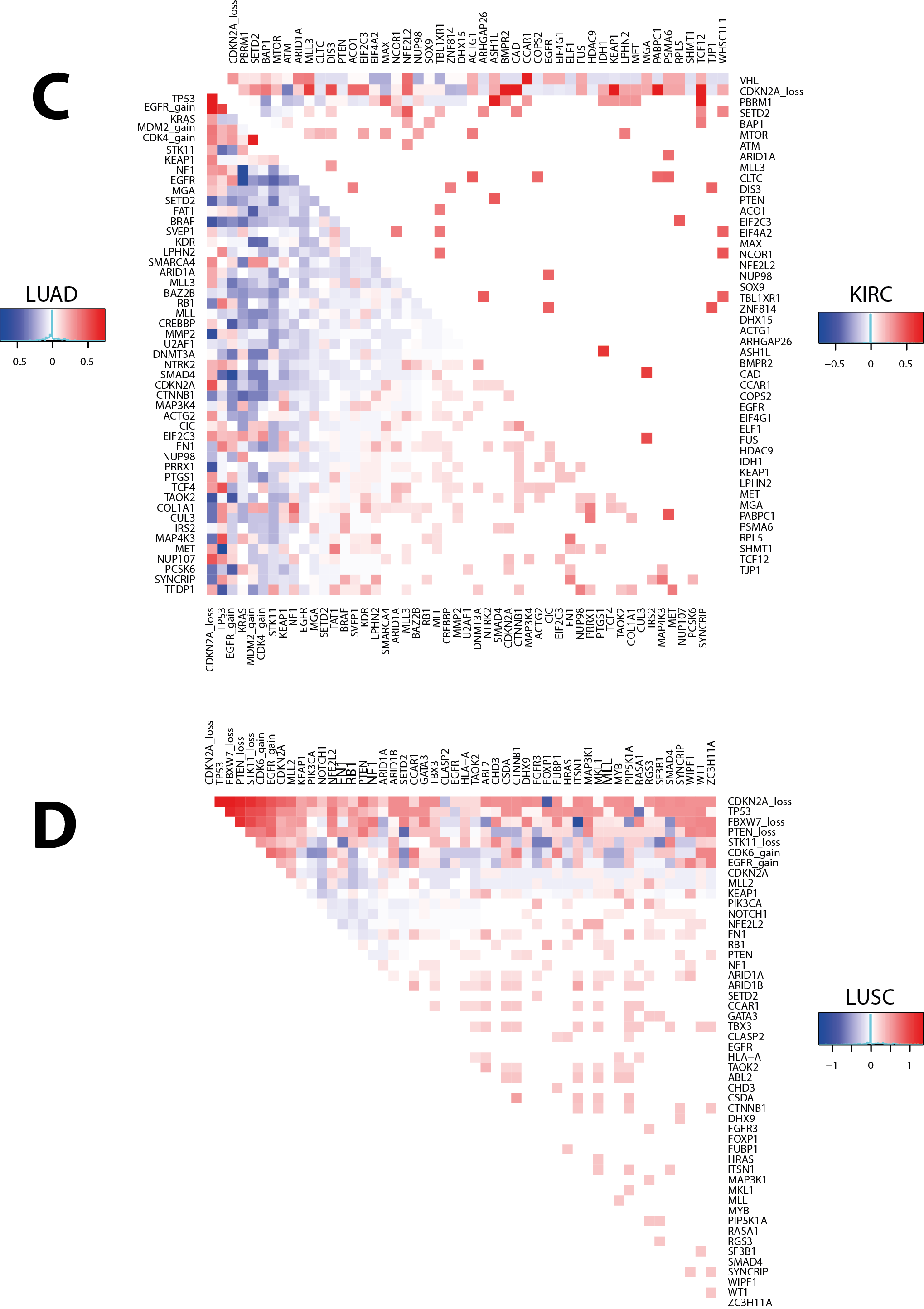


**Supplementary Figure 4 – Epistatic interactions (continued).**


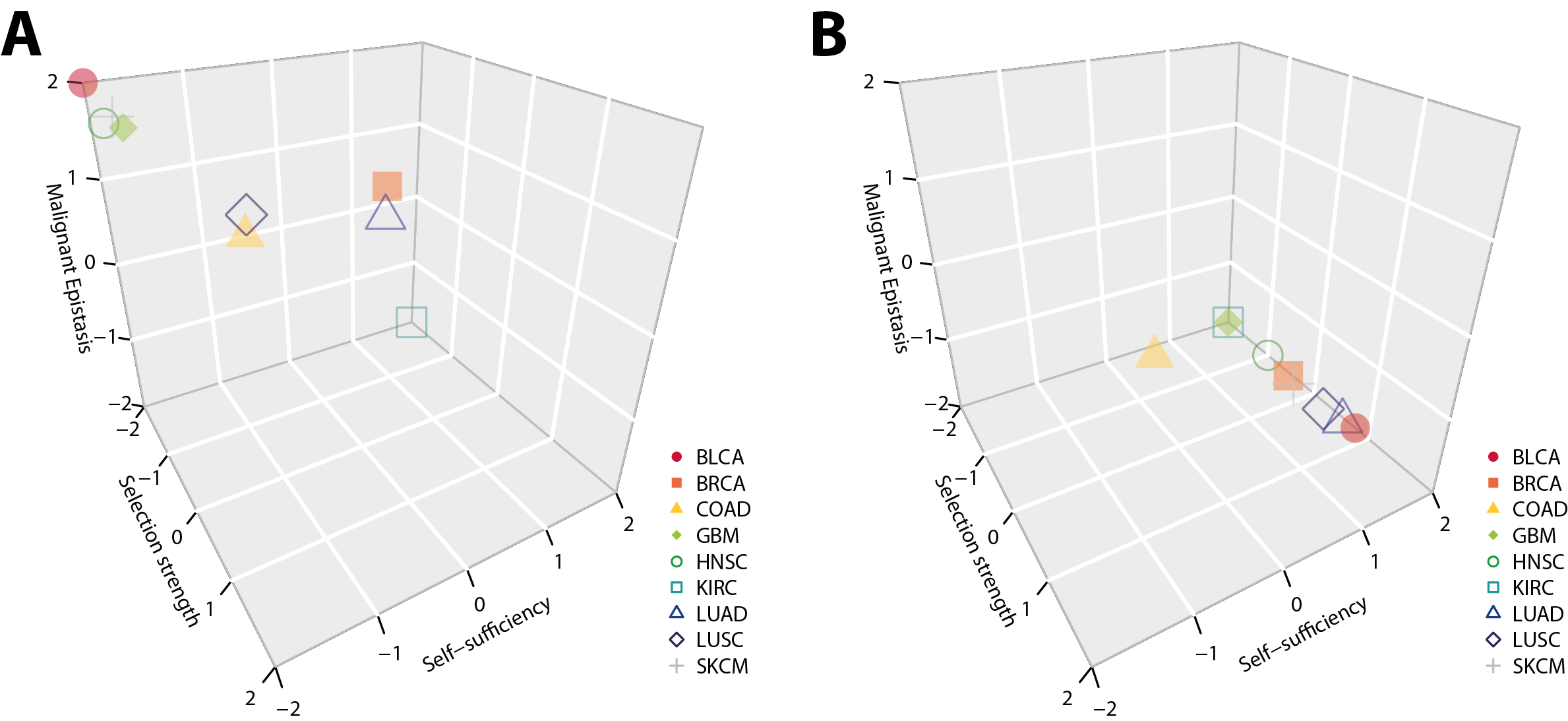


**Supplementary Figure 5 – Separate models weight optimization, alternative angle.** Optimal weights found for selective advantage, self-sufficiency and epistatic interactions in the “separate” models (log10 scale). A) separate_mean; B) separate_prod.

**
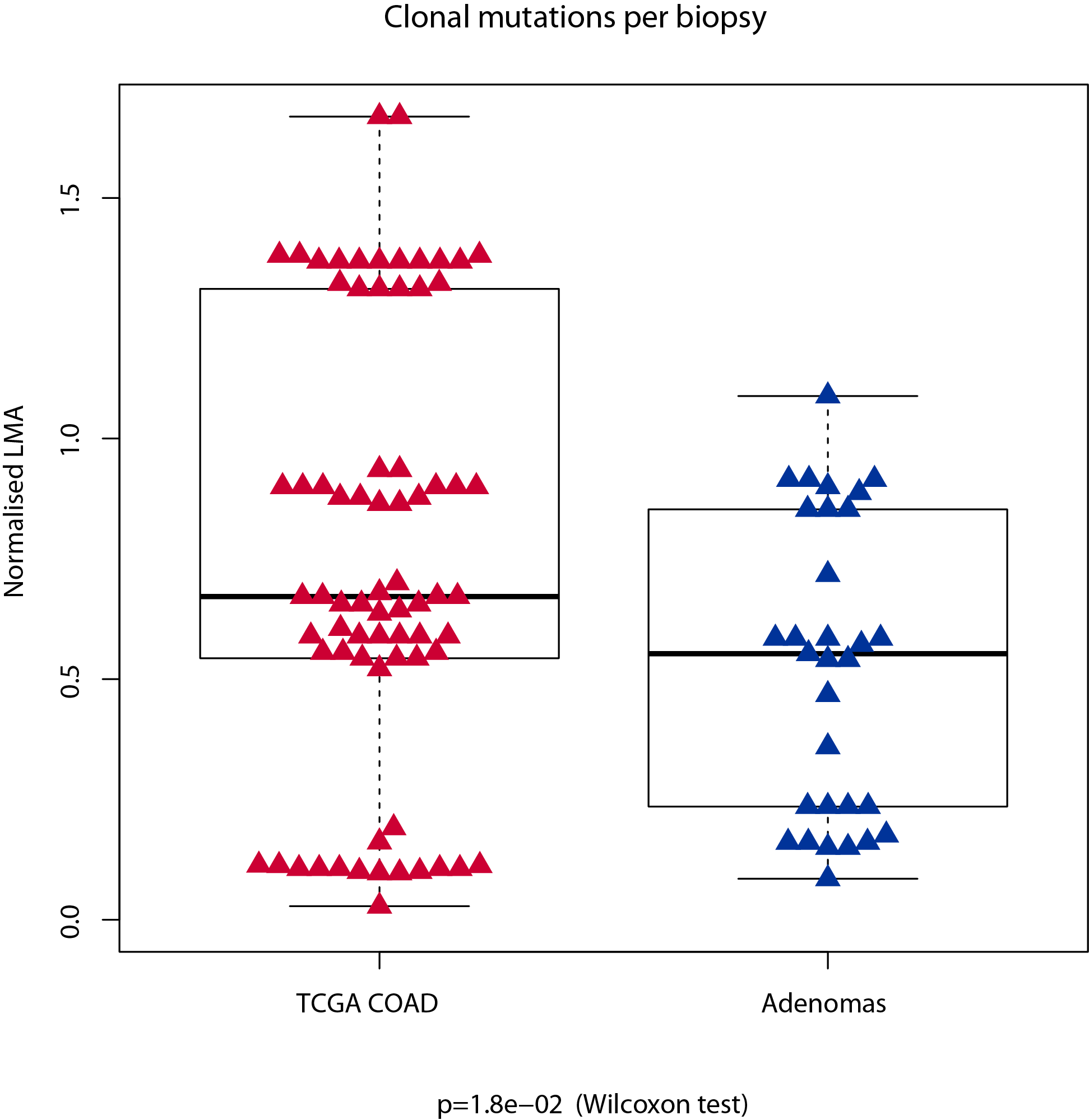
**

**Supplementary Figure 6 – LMA scores of carcinoma and adenoma biopsies.** Normalised LMA scores of multiple biopsies from 11 11 colorectal carcinomas (left) and 9 adenomas (right).


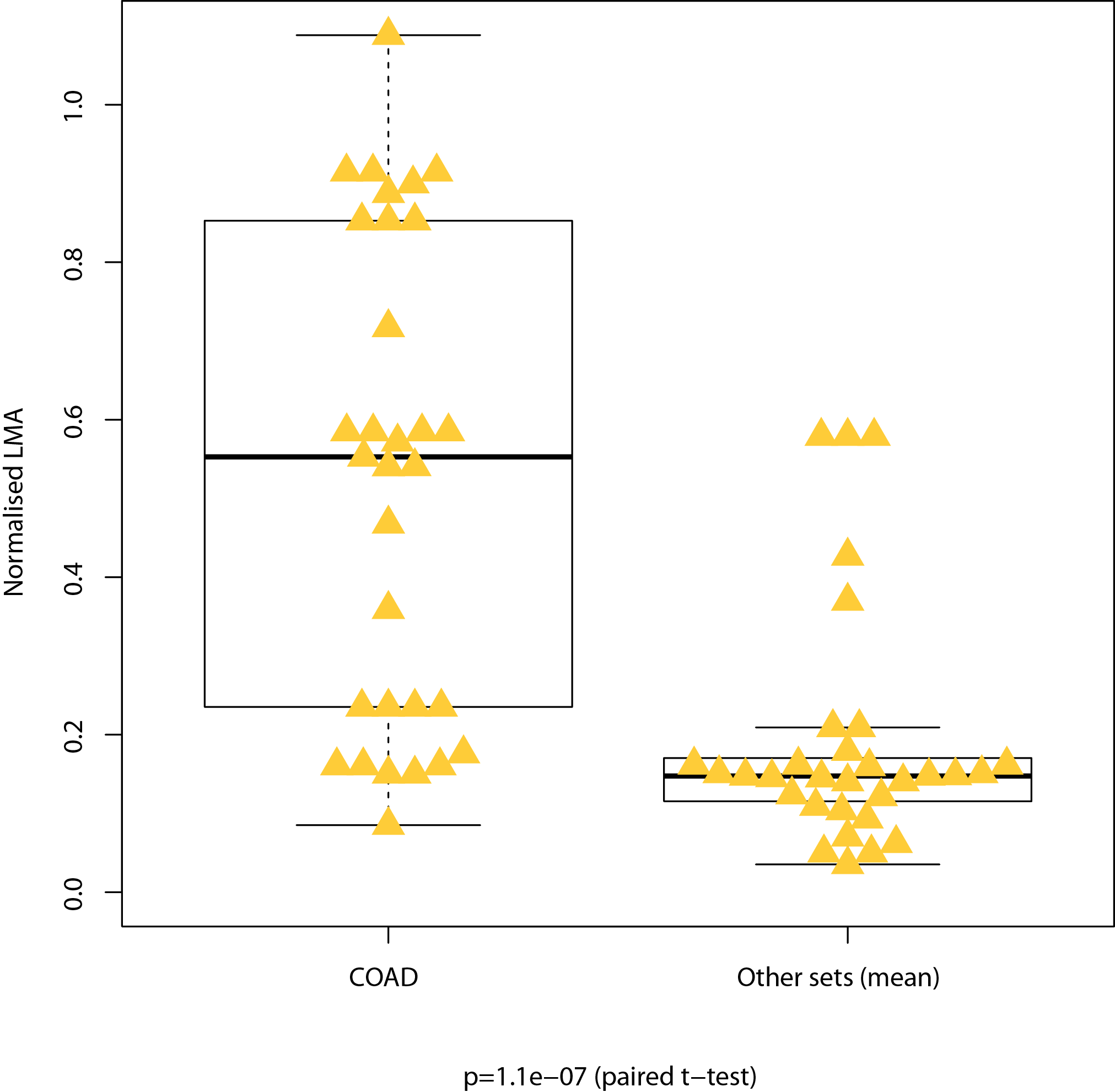


**Supplementary Figure 7 – LMA scores of adenoma biopsies in the colorectal adenocarcinoma (COAD) landscape and other landscapes.** Normalised LMA scores of adenomas in the COAD landscape (left) and in the 8 other landscapes on average (right).


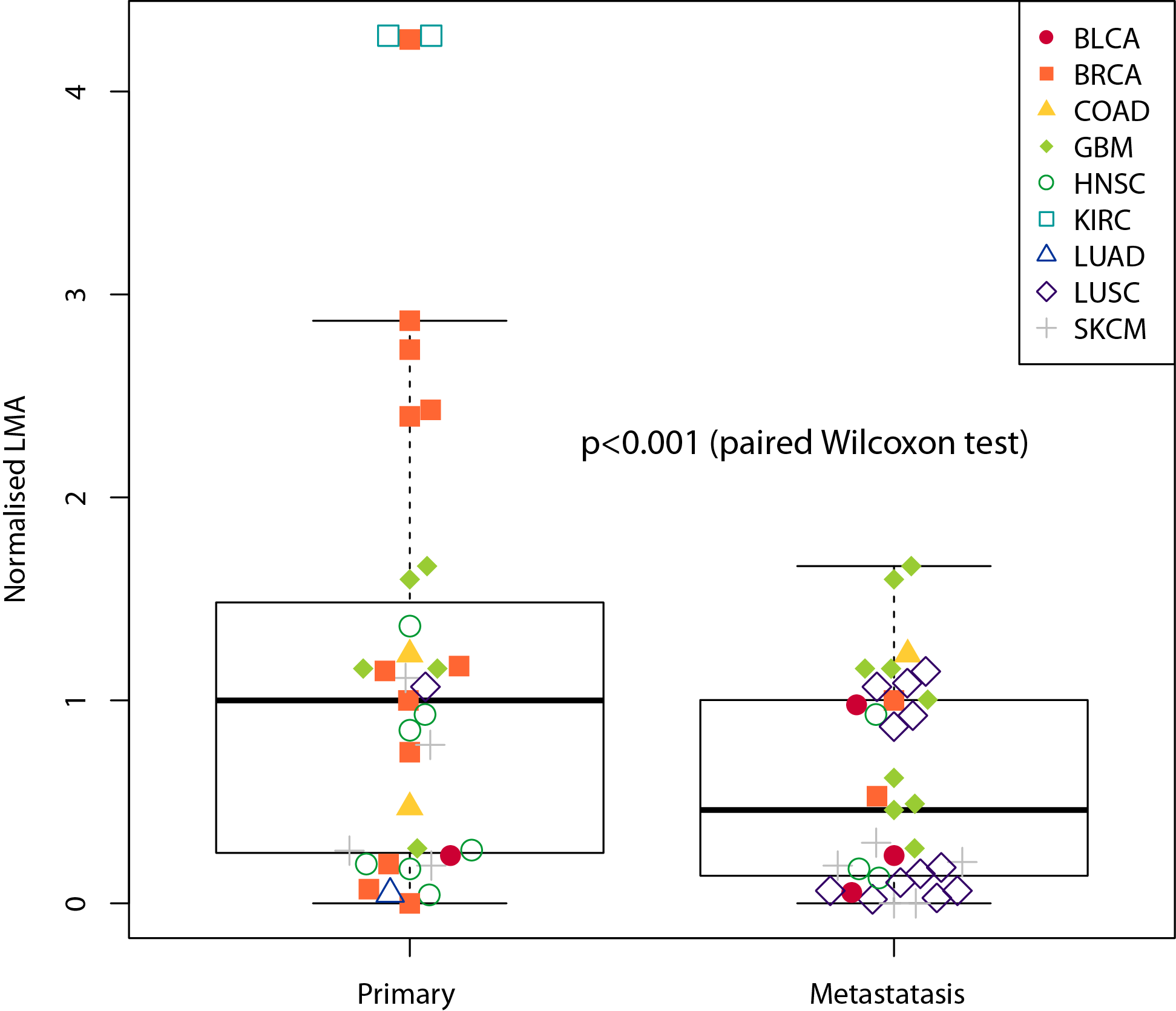


**Supplementary Figure 8 – LMA scores of metastatic lesions in the landscapes of the primary and metastatic sites.** Normalised LMA scores of 35 lesions with landscapes existing for both primary and metastatic site. Left, LMA in the primary site’s landscape; right, LMA in the metastatic site’s landscape. The LUSC landscape was used to estimate the Local Malignant Adaptation of lung metastatic lesions, as opposed to LUAD in the main text figure.

**
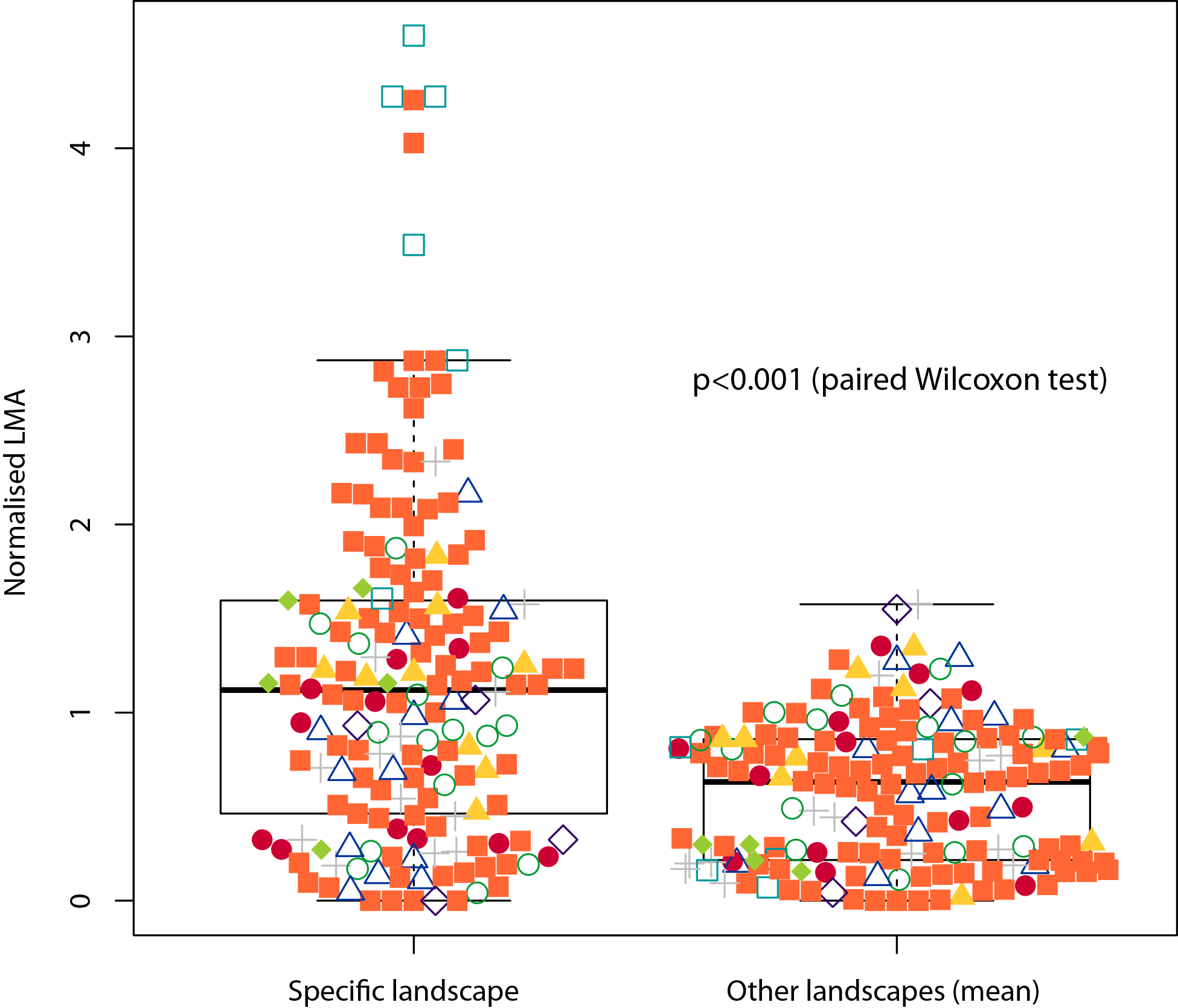
**

**Supplementary Figure 9 – LMA scores of metastatic lesions in the primary landscape compared to other landscapes.** Normalised LMA score of 170 lesions with a primary site landscape in this specific landscape (left), or in the other 8 landscapes on average (right). The LUSC landscape was used to estimate the Local Malignant Adaptation of lung metastatic lesions, as opposed to LUAD in the main text figure.

**
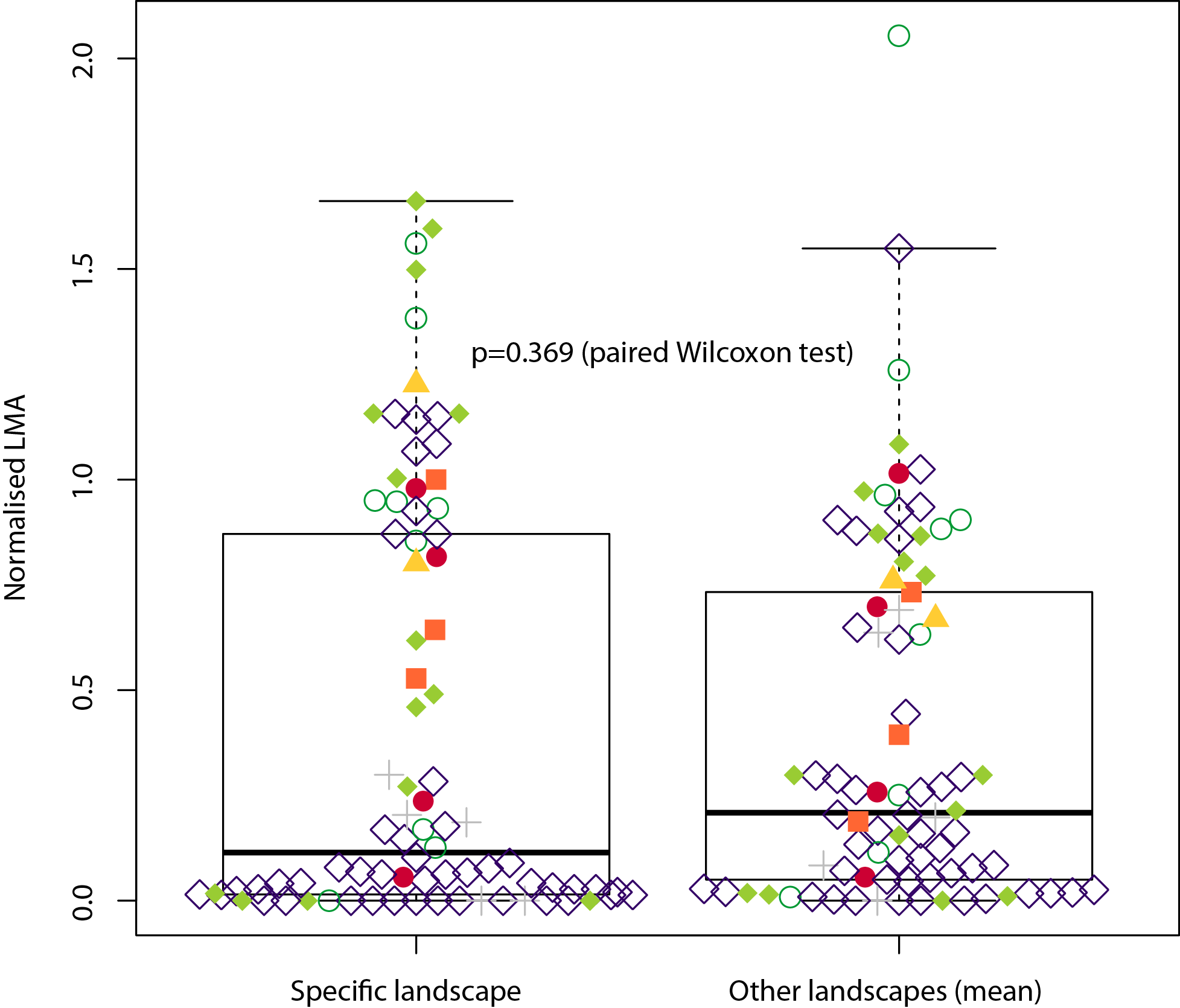
**

**Supplementary Figure 10 – LMA score of metastatic lesions in the metastatic landscape compared to other landscapes.** Normalised LMA score of 82 lesions with a metastatic site landscape in this specific landscape (left), or in the other 8 landscapes on average (right). The LUSC landscape was used to estimate the Local Malignant Adaptation of lung metastatic lesions, as opposed to LUAD in the main text figure.
